# Percutaneous Computed Tomography (CT) Fluoroscopy-Guided Biopsy of the Spleen Using Fibrin Glue as a Sealant

**DOI:** 10.3390/diagnostics14020162

**Published:** 2024-01-11

**Authors:** Laura Segger, Markus Herbert Lerchbaumer, Federico Collettini, Bernd Hamm, Florian Nima Fleckenstein, Uli Fehrenbach, Bernhard Gebauer, Timo A. Auer

**Affiliations:** 1 Department of Radiology, Campus Virchow-Klinikum, Charité—Universitätsmedizin Berlin, 13353 Berlin, Germany; federico.collettini@charite.de (F.C.); bernd.hamm@charite.de (B.H.); florian.fleckenstein@charite.de (F.N.F.); uli.fehrenbach@charite.de (U.F.); bernhard.gebauer@charite.de (B.G.); timo.auer@charite.de (T.A.A.); 2 Department of Radiology, Campus Mitte, Charité—Universitätsmedizin Berlin, 10117 Berlin, Germany; markus.lerchbaumer@charite.de; 3 Berlin Institute of Health (BIH), Anna-Louisa-Karsch-Straße 2, 10178 Berlin, Germany

**Keywords:** percutaneous image-guided biopsy, biopsy of the spleen, CT-guided intervention, splenic lesions

## Abstract

Some authors consider the risk of bleeding an absolute contraindication to percutaneous image-guided splenic puncture. While splenic punctures are mainly performed at specialized centers, no technique for the closure of the puncture tract has been broadly established. The aim of this study was to investigate the effectiveness and safety of a percutaneous image-guided biopsy of the spleen using fibrin glue to plug the tract. A total of 27 requests for splenic image-guided interventions were identified between 2010 and 2021 and considered for inclusion in our retrospective single-center study. Seven patients needed to be excluded, which left twenty patients who underwent a percutaneous computed tomography (CT) fluoroscopy-guided biopsy of a splenic lesion during this period. In all patients, a 17G coaxial needle with an 18G core biopsy needle was used. Diagnostic adequacy and accuracy were evaluated, and complications were classified using the CIRSE classification system for adverse events. Diagnostic adequacy was 100% (20/20), and a median of four samples were collected. Diagnostic accuracy was 80% (16/20). The four off-target samples included one inconclusive finding and three samples of regular spleen tissue. The overall complication rate was 5% (1/20). No mild (grade 1–2) or moderate (grade 3–4) complications occurred. One severe (grade 5–6) complication occurred. Although controversial and potentially high-risk, diagnostic percutaneous biopsies of the spleen appear to be relatively safe with the use of fibrin glue to seal the tract.

## 1. Introduction

Focal splenic lesions that cannot be classified by computed tomography (CT) or magnetic resonance imaging (MRI) are rare. Yet, a diagnostic work-up can be challenging in these cases, and in some cases, histological results are required for further work-up [1,2]. In the past, patients underwent splenectomy to establish the correct diagnosis by histology [3]. Most splenic masses, such as cysts and hemangiomas, are benign lesions, and the most common malignant lesions, like manifestations of lymphoma or splenic metastasis, are usually identified in systemic reviews [4,5]. Only rarely do patients require an operation and splenectomy. Therefore, a surgical procedure would be unnecessary and will not treat systemic disease. Because of the high blood flow to the spleen, biopsy used to be rather uncommon due to the inherent high risk of bleeding, with studies reporting a high rate of complications [6,7]. But as an operation with splenectomy is also not without its risks, it can be an advantage to perform a percutaneous biopsy and spare patients a splenectomy [8]. Since then, several studies and meta-analyses have been performed on the percutaneous image-guided biopsy of the spleen and the risk of bleeding [9,10]. There is an ongoing debate as to which biopsy needles are best in this regard, and different studies have been published comparing different needles [11,12]. Additionally, endoscopic ultrasound-guided tissue acquisition has been introduced as another alternative [13].

There have already been studies of laparoscopic splenectomy and the usage of fibrin glue to reduce the risk of bleeding [14,15]. A few studies have shown that liver biopsy with fibrin glue to plug the tract can lower the rate of bleeding complications, especially in patients with an increased risk of bleeding due to coagulation disorders, platelet dysfunction, or ascites [16,17]. It is an open question if this is also a favorable technique for the biopsy of the spleen. Some investigators consider the risk of bleeding in percutaneous image-guided splenic punctures an absolute contraindication [18].

Yet, splenic punctures are mainly performed at specialized centers, but no technique that closes the puncture tract has been broadly established. Therefore, this study aims to present a technique to close the puncture tract and analyze the complications occurring in splenic biopsy when fibrin glue is used as a sealant.

## 2. Materials and Methods

This retrospective study was approved by the institutional review board.

### 2.1. Study Population

The investigation period was from 2010 to 2021 and included all requested CT-guided splenic biopsies with the usage of fibrin glue as a sealant. Exclusion criteria were no received images, missing clinical data, and no notification of the usage of fibrin glue as a sealant.

General and clinical information on the 20 study patients was retrieved from the institutional radiology information system (see Supplementary Material). The data were collected in a register and included general patient information. There were no patients with missing clinical data and/or missing image datasets. Information on complications was obtained by retrieving findings of follow-up examinations performed during hospital stays from the hospital information system.

**Figure 1:**
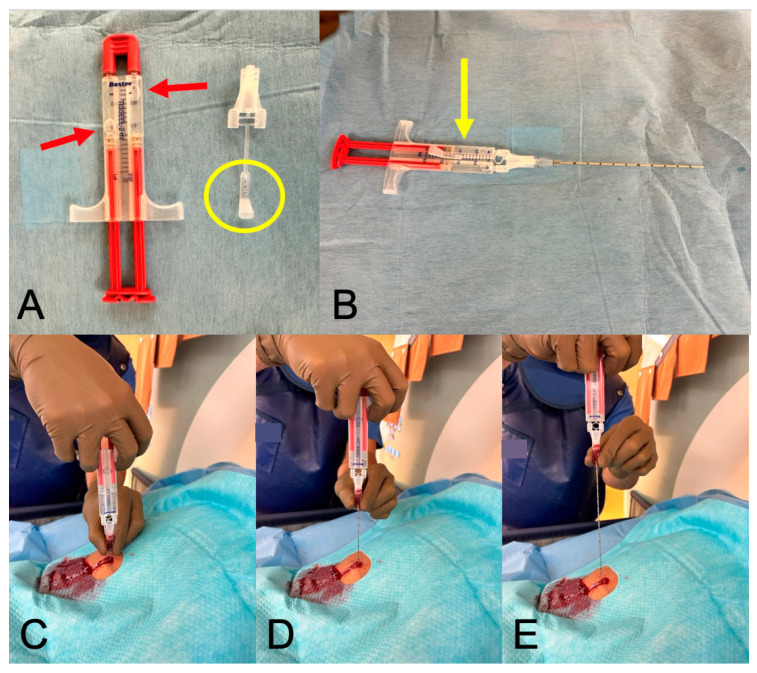
Fibrin glue sealant—practical implementation. Legend: In (**A**), the thawed fibrin two-component adhesive is displayed, with two air bubbles visible in the sealed tubes (red arrows). Adjacent to it is the attachment cap and its lug (yellow circle). (**B**) depicts the adhesive with the attachment securely locked (yellow arrow), along with the 17G coaxial needle attached to it. (**C**–**E**) illustrate the process of gluing the puncture channel: the needle is gradually withdrawn while a continuous stream of 2 mL of glue is being administered.

An interventional radiologist with more than 15 years of experience supervised each biopsy. After local anesthesia, percutaneous biopsy was performed using a 17G coaxial needle with an 18G core biopsy needle (Bard Peripheral Vascular, Inc., Tempe, AZ, USA). After tissue sampling, the biopsy tract was filled with an adhesive composed of human thrombin and fibrinogen as a sealant (TISSEL/TISSUCOL, BAXTER Sachwert GmbH & Co. KG, Berlin, Germany); Figure 1. Patients were told to lie on their left side and stay in bed for at least two hours, followed by a full blood count to check hemoglobin levels. An ultrasound examination was scheduled for the following day.

### 2.2. Outcome Measures

**Figure 2:**
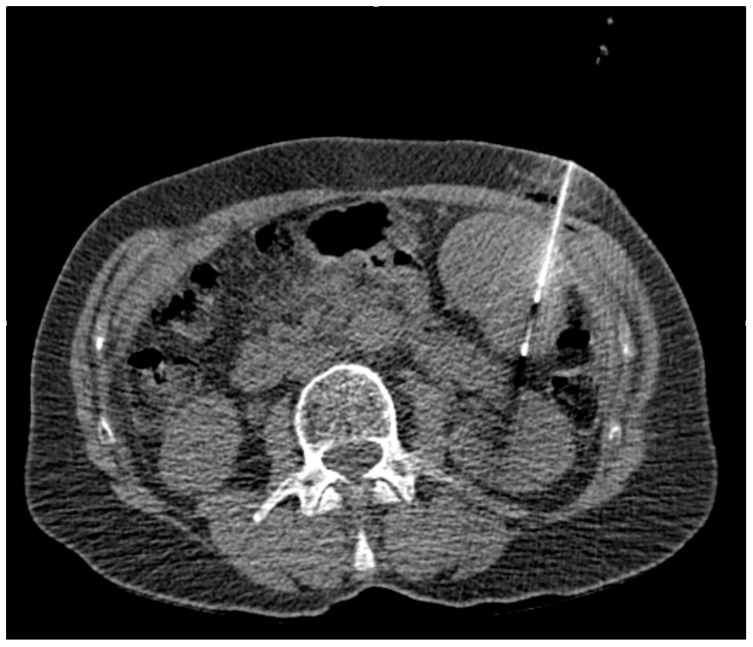
CT-guided spleen biopsy.

The primary aim of our retrospective study was to systematically characterize splenic biopsy in terms of success, types of complications, and complication rates using fibrin glue as a sealant. Diagnostic adequacy was defined as obtaining at least one representative tissue sample and an accompanying fluoroscopic image documenting the extended needles in the spleen or lesion; Figure 2.

Diagnostic accuracy was defined as confirmation of the suspected diagnosis or detection of non-splenic tissue in the biopsy sample. Complications were graded using the CIRSE Quality Assurance Document and Standards for Classification of Complications: The CIRSE Classification System [19]. Grades 5 and 6 were classified as severe complications, grade 3 and 4 as moderate complications, and grades 1 and 2 as mild complications. The duration of the CT-guided biopsy procedures was recorded as the interval between the first i-sequence on CT and the final i-sequence. All data were analyzed using Microsoft Excel (Version 16.59).

## 3. Results

**Figure 3:**
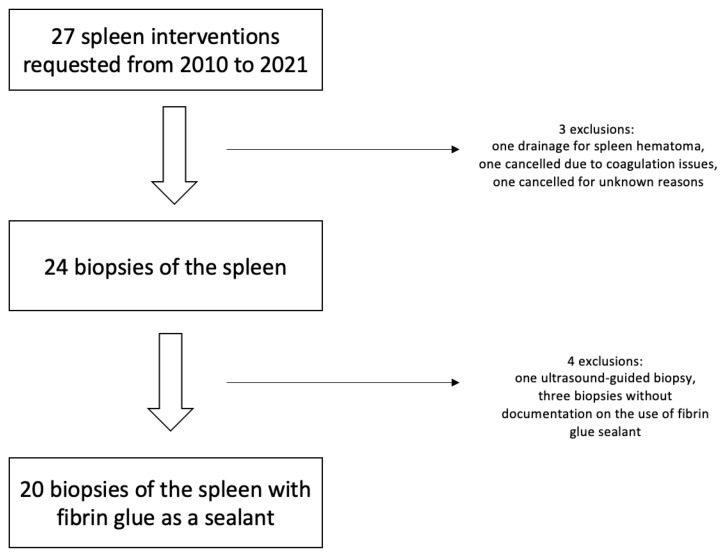
Flowchart of patient inclusion.

A total of 27 requested splenic interventions were identified between 2010 and 2021; Figure 3. Three requests were excluded: one was a drainage of a hematoma, one was canceled because of coagulation issues, and one was not carried out for unknown reasons. The remaining 24 patients with unclear splenic lesions underwent biopsy and were considered for this study. Twenty-three patients underwent CT fluoroscopy-guided biopsy, of which twenty were carried out with a fibrin glue as a sealant. One patient had an ultrasound-guided biopsy with fibrin glue sealant. The ultrasound-guided biopsy and the three biopsies without fibrin glue were excluded.

The final study population consisted of twelve men and eight women with a median age of 65 years. The median time required for the biopsy procedure was 19 min, and the median radiation dose per procedure was 316 mGy-cm (dose-length product); Table 1.

The overall success rate of the biopsy was 100% (20/20). A histologic analysis was possible in 100% (20/20) of cases and yielded a diagnosis in 80% of patients (16/20). Five percent (1/20) were inconclusive, and 15% (3/20) showed normal spleen tissue. The most common histologic result was B-cell lymphoma, which was found in 50% of the study patients. Rare diagnoses included Langerhans cell sarcoma or angiosarcoma. An overview of all histologic diagnoses is presented in Table 2.

One of the patients with normal splenic tissue in the first biopsy underwent a repeat biopsy with inconclusive histologic findings. Regular CT follow-up for two years showed unchanged splenic lesions in this patient. The second patient with initially normal histologic results was re-biopsied using ultrasound guidance. The second histologic examination showed a littoral cell angioma, a benign tumor of the spleen derived from the red pulp sinuses [20]. The third patient with normal histology had known multiple myeloma (type IgG lambda) and splenomegaly with a craniocaudal extent of 22 cm and was biopsied for suspected amyloidosis. Histology revealed no sign of amyloidosis. An endoscopic intestinal biopsy was also negative for amyloidosis. No further information on this patient was available from the documentation system, suggesting that the patient was lost to further follow-up.

Most of the patients were referred for a biopsy from the Department of Internal Medicine (95.0%, 19/20); among them, 75% (15/19) were from the hemato-oncology ward, 15% (3/19) were from the infectious diseases ward, and 5% (1/19) were from the nephrology ward. The remaining patient was referred from the gynecology ward. The splenic lesion, in this case, turned out to be a metastasis of an estrogen receptor-positive, HER2-negative carcinoma.

### Complications

The overall complication rate was 5% (1/20). None of the patients had mild (grade 1–2) or moderate (grade 3–4) complications. One severe complication (grade 5–6) rated as CIRSE grade 6 occurred, which was severe bleeding and was treated by selective embolization.

## 4. Discussion

Although a biopsy of the spleen should only be obtained for splenic lesions that require further workup, a diagnostic percutaneous biopsy of the spleen using fibrin glue as a sealant appears to be a relatively safe and effective procedure.

With differing emerging guidelines on how to diagnose splenic lesions, we noted an increasing interest in splenic biopsy in our hospital before the COVID-19 pandemic. A drop in the demand between 2019 and 2021 seems to be in line with an overall delay in cancer diagnosis during the pandemic [21,22]. Additionally, the management of incidental splenic lesions has changed in the past couple of years, as the first “white paper” of the American College of Radiology recommends a follow-up for incidental splenic masses over >1 cm, while another more recent study by Siewert et al. even concludes that a splenic mass at CT does not need further work-up in patients without left upper-quadrant pain and without a history of malignancy regardless of the size of the mass [1,23]. These studies explain the limited indications for splenic biopsy and the rather small number of cases, even in a tertiary referral hospital. Still, there remain patients with symptoms or known malignancy who can benefit from a biopsy rather than a splenectomy. As our results show, the most common diagnosis is B-cell lymphoma, which usually does not need a splenectomy for therapy. A CT-guided biopsy has several advantages over a splenectomy; it is much less invasive, faster to perform (a mean duration of 19 min in our study versus 44 min for splenectomy), and has a lower overall risk, not to mention the cost-effectiveness [24]. In comparison to the endoscopic ultrasound-guided tissue acquisition, which is summarized by Lisotti et al., the CT-guided biopsies have the advantage of obtaining sufficient material to perform immunohistochemistry and, therefore, allow the diagnosis of lymphoma subtypes while having a similar complication rate (5% in CT-guided biopsy and 4.7% in endoscopic biopsy) [13]. Most studies reported in the literature investigated biopsies performed with ultrasound guidance. It is important to mention that there are numerous research articles comparing the cost-effectiveness of ultrasound-guided biopsy and CT-guided biopsy, demonstrating that ultrasound-guided biopsies offer greater cost-effectiveness [25,26,27]. The decision to utilize CT is based on the extensive expertise we have developed in this field over the years. It is noteworthy that 90% of all organ punctures at our clinic are CT-guided. The literature on CT-guided biopsies of the spleen is sparse, and only one study with seven patients investigated the use of a gel-based preparation to seal the biopsy tract [28,29].

Special attention needs to be brought to the needle gauge. Bigger needles usually result in a higher diagnostic accuracy but also tend to have a higher risk of bleeding [9]. Liang et al. compared the use of 18G and 21G needles for spleen biopsy and found a higher diagnostic accuracy for 18G needles without a difference in complications [11]. The 80% diagnostic success rate achieved in our study is in the range of 75–100% reported in the literature for 18G needles, although the data for using 18G are sparse, with only small patient groups under ultrasound guidance [6]. Taking four samples, we achieved a comparably high diagnostic accuracy [6,11]. Overall, only one severe complication occurred in our patients.

Three cases—the one with inconclusive histology and two of three cases with normal splenic tissue on initial biopsy—remained unsolved. In the remaining case with initially normal histology, a repeat biopsy with ultrasound guidance yielded the rare diagnosis of littoral cell angioma. As the group of 20 patients is small, a single complication corresponds to a complication rate of 5%, which seems to be rather high. Even though no mild or moderate complication occurred, bleeding requiring angiographic embolization was reported in one case. The patient developed severe coagulation issues, and a major part of the spleen was infarcted through the embolization. Several days later, the decision to perform an emergency splenectomy was made in that case. Subsequently, the differential diagnosis of Stevens–Johnson syndrome was made, and weeks later, the patient died due to multiple system organ failure not related to the bleeding complication.

Our study has some limitations, including its retrospective, single-center design with the analysis of a preselected patient population. Additionally, a Selection Bias in the results cannot be ruled out due to the high percentage of B-cell lymphoma in the histologic results. Nevertheless, the study addresses an important topic since there are little data on the biopsy of the spleen with fibrin glue as a sealant. Another limitation is the short follow-up period. Nevertheless, our results suggest that a spleen biopsy with fibrin glue sealant is a comparably safe and effective method to obtain adequate tissue samples for histologic diagnosis.

## 5. Conclusions

A total of 20 patients who underwent CT-guided spleen biopsy using a 17G coaxial needle system with an 18G core biopsy needle and fibrin glue as a sealant for the puncture tract were analyzed. In conclusion, our findings show that a biopsy of the spleen with fibrin glue as a sealant is a rather safe and effective procedure. However, this conclusion needs to be confirmed prospectively and in larger study populations.

## Figures and Tables

**Table 1:** General characteristics of the study population.

Study Population	
Age in years (median + IQR)	65 + 16.5 years
Male-to-female ratio	12 to 8
Median biopsy duration	19 min
Median radiation dose per procedure (dose-length product)	316 mGy-cm (DLP)

**Table 2:** Histologic results.

*n*=	Histologic Results
10	B-cell lymphoma
1	Extramedullary hematopoesis
1	Normal spleen tissue, slight increase in histiocytes (= inconclusive)
1	Infarction
1	Metastasis of an estrogen receptor-positive, HER2-negative carcinoma
1	Dendritic/histiocytic sarcoma (Langerhans cell sarcoma)
1	Angiosarcoma
1	Purulent infection
3	Regular spleen tissue

## Data Availability

The data that support the findings of this study are not openly available due to reasons of sensitivity and are available from the corresponding author upon reasonable request. Data are located in controlled access data storage at Campus Virchow Klinikum of the Charité Universitätsmedizin Berlin, Germany.

## Supplementary Materials

The following supporting information can be downloaded at: https://www.mdpi.com/article/10.3390/diagnostics14020162/s1, Summary of patient data.
