# Supplementary material for: Percutaneous Computed Tomography (CT) Fluoroscopy-Guided Biopsy of the Spleen Using Fibrin Glue as a Sealant
Source: Diagnostics (Basel). 2024 Jan 11;14(2):162. doi: 10.3390/diagnostics14020162 (PMC10814976; doi:10.3390/diagnostics14020162)
Supplement: Supplementary file 1 [file diagnostics-14-00162-s001.zip › diagnostics-2771380-supplementary.pdf]

Patient	Sex	Age in years	First i-sequence	Last i-sequence	Radiation dose mGycm (DLP)	Diagnostic adequacy	Sample number	Histologic results	Bleeding
1	M	36	13:15	13:28	472	yes	6	Infarction	0
2	M	74	10:46	10:55	451	yes	5	B-cell lymphoma	0
3	F	52	09:49	10:01	318	yes	2	B-cell lymphoma	0
4	M	76	15:56	16:09	184	yes	4	Extramedullary hematopoesis	0
5	F	63	13:19	13:54	266	yes	5	Metastasis of an estrogen receptor-positive, HER2-negative carcinoma	0
6	M	22	12:37	13:04	212	yes	7	Dendritic/histiocytic sarcoma (Langerhans cell sarcoma)	0
7	M	69	15:31	15:40	222	yes	4	B-cell lymphoma	0
8	F	33	11:41	12:00	254	yes	3	B-cell lymphoma	1
9	M	49	13:37	14:06	465	yes	3	Regular spleen tissue	0
10	F	71	15:21	15:37	161	yes	4	B-cell lymphoma	0
11	F	65	13:24	13:36	314	yes	5	B-cell lymphoma	0
12	F	51	16:05	16:24	379	yes	3	Purulent infection	0
13	F	68	14:19	15:22	171	yes	1	Angiosarcoma	0
14	M	65	15:56	16:20	586	yes	3	Regular spleen tissue	0
15	M	65	08:53	09:27	373	yes	7	Normal spleen tissue, slight increase in histiocytes (=inconclusive)	0
16	M	58	12:08	12:27	815	yes	x	B-cell lymphoma	0
17	M	79	11:21	11:41	363	yes	4	B-cell lymphoma	0
18	M	63	16:32	16:47	197	yes	4	Regular spleen tissue	0
19	M	65	13:38	13:57	324	yes	4	B-cell lymphoma	0
20	F	65	13:24	13:36	314	yes	5	B-cell lymphoma	0

Supplement 1. Summary of patient data.
